# Serious Adverse Events in the Canadian Registry of Children Receiving Palivizumab (CARESS) for Respiratory Syncytial Virus Prevention

**DOI:** 10.1371/journal.pone.0134711

**Published:** 2015-08-03

**Authors:** Jinghan Jenny Chen, Parco Chan, Bosco Paes, Ian Mitchell, Abby Li, Krista L. Lanctôt, CARESS investigators

**Affiliations:** 1 Medical Outcomes and Research in Economics (MORE) Research Group, Sunnybrook Health Sciences Centre, University of Toronto, Toronto, Ontario, Canada; 2 Department of Pediatrics, McMaster University, Hamilton, Ontario, Canada; 3 Department of Pediatrics, University of Calgary, Calgary, Alberta, Canada; University ofTennessee Health Science Center, UNITED STATES

## Abstract

**Objectives:**

To evaluate the safety and tolerability of palivizumab for RSV prophylaxis in high-risk children in everyday practice.

**Methods:**

High-risk children prophylaxed against RSV infection were recruited into a prospective, observational, Canadian RSV Evaluation Study of Palivizumab (CARESS) registry with active, serious adverse event (SAE) monitoring from 2008 to 2013. SAE reports were systematically collected and assessed for severity and relationship to palivizumab. Data were analyzed by Chi-square or Fisher Exact Tests to examine group differences in proportions.

**Results:**

13025 infants received 57392 injections. Hospitalizations for respiratory-related illness (RIH) were reported in 915 patients, and SAEs other than RIH were reported in 52 patients. Of these, 6 (0.05%) patients had a total of 14 hypersensitivity reactions that were deemed possibly or probably related to palivizumab (incidence: 2.8 per 10,000 patient-months). The SAEs of 42 patients were assessed as not related to palivizumab. SAEs in the remaining 4 patients were not classifiable as their records were incomplete. There were no significant demographic predictors of SAE occurrence.

**Conclusions:**

Under active surveillance, a small proportion of infants in the CARESS registry experienced SAEs that had a potential relationship with palivizumab and these appeared to be unpredictable in terms of onset. Palivizumab appears to be a safe and well-tolerated antibody for RSV prophylaxis in high-risk children in routine practice.

## Introduction

Respiratory syncytial virus (RSV) is a highly ubiquitous viral respiratory pathogen in children[1–3] with infections occurring seasonally, peaking during the winter months[4]. RSV infection involving the upper airway may progress to lower respiratory tract disease, which is associated with recurrent wheezing and asthma in childhood[5–9] and significantly increases health resource utilization[10]. In Canada, Langley et al. estimated that RSV-associated illness costs US$18 million annually, with inpatient hospital care and ambulatory expenditure as being the top contributors[11]. Palivizumab has been studied extensively in children who are at-risk for RSV-related hospitalizations, and has been shown to significantly reduce both the incidence of hospitalizations and duration of hospital stay [12, 13].

Palivizumab is a composite monoclonal antibody with 95% human and 5% murine amino acid sequences and a molecular weight of approximately 148,000 Daltons[14–17]. Despite humanization and the minimal homology with the mouse antibody, palivizumab still has immunogenic potential and can induce anti-palivizumab antibodies, which could alter its therapeutic efficacy and incite hypersensitivity reactions [18–20]. In 2002, the manufacturer of palivizumab reported an incidence of 2 anaphylactic cases in an estimated population of 400,000 patients (<1 per 100,000 patients) based on four seasons of world-wide post-marketing data [21]. While those data are reassuring, the numerator relies on voluntary reports submitted to a passive surveillance system, which may lead to an underestimation of adverse events (AE), and the denominator is approximated. AEs associated with palivizumab were also assessed as part of the Phase I-III clinical trials [12–13, 22–24] and in follow-up studies [25–31]. However, sample sizes in those studies were too small to detect rare events and the populations under surveillance may not reflect real world outcomes in other at-risk infants who receive prophylaxis (e.g., neuromuscular impairments, immunocompromise and cystic fibrosis) [12–13, 22–31]. Although overall mortality was previously reported by Mitchell et al, it was assessed in a smaller sample of subjects who had received palivizumab without an in-depth analysis of the documented AEs [32]. As such, data are lacking on the incidence and characteristics of serious adverse events (SAEs) associated with palivizumab.

The primary objective of this study was to describe SAEs in children recruited into the Canadian RSV Evaluation Study of Palivizumab (CARESS) database, who received palivizumab during the 2008–2013 RSV seasons. The secondary aim was to examine if any demographic risk factors were associated with an SAE occurrence. In addition, palivizumab discontinuations were assessed to quantify tolerability and evaluate whether SAEs were associated with termination of prophylaxis.

## Methods

### Palivizumab Administration

Palivizumab is injected intramuscularly at a dose of 15 mg/kg of body weight. An interval of 16–35 days between the first and second injection and 25–35 days for subsequent injections has been adopted provincially across Canada in order to target appropriate therapeutic serum palivizumab levels for RSV prevention. Patients are expected to receive an injection on a monthly basis from the time of enrolment to the end of the RSV season which averages 4 to 5 injections per season dependent on when the first palivizumab injection was administered [33]. The length of the RSV season is site-specific and the starting date is determined by local clinicians, based on historic patterns of RSV epidemiology and community based rates of infection. Similarly the season offset is also determined by local clinicians usually after 2 consecutive 7-day periods with 1 or no RSV hospital admissions [34].

### Canadian RSV Evaluation Study of Palivizumab (CARESS)

CARESS is a prospective, longitudinal, observational, open-cohort study of pediatric patients, who received at least one injection of palivizumab across 32 Canadian sites during any of the RSV seasons from 2008 to 2013. Written, informed consent is obtained from parents or legal guardians before patients are enrolled into the study. An electronic case report form is used for all stages of data collection to ensure consistency. Information regarding demographics, medical history, neonatal course, and palivizumab administration is collected at enrolment. Subsequent monthly interviews are conducted by research nurses beginning when consent is obtained, each occurring within 4 weeks of the previous palivizumab injection. The interviews are conducted until 30 days after the patient’s last injection. In these monthly interviews, data on: palivizumab administration, changes in baseline data, hospitalization for respiratory illness (RIH), diagnostic tests for RSV-related infection, reasons attributing to the discontinuation of palivizumab, and clinical evidence of AEs are collected from the patients’ parents or guardian. In the event of a RIH, with additional parental/legal guardian consent, the research nurse reviews the relevant hospital records and completes a corresponding hospitalization form.

### Ethics Statement

Written informed consent was obtained from parents or legal guardians of subjects by study nurses at each site. Consent was obtained according to standard operating procedures at each site, and was approved by the site's research ethics board. The study was reviewed and approved by each site’s respective research ethics board for compliance with the Declaration of Helsinki principles and the standards of Good Clinical Practice. Please see endnote of the article for a list of each specific site’s research ethics board***. The CARESS registry is registered under ClinicalTrials.gov Identifier: NCT00420966.

### Definitions and reporting of adverse events

An adverse event (AE) was defined as any unexpected occurrence in a patient after palivizumab was administered, that may or may not have a causal relationship with the drug. Treatment-emergent AEs refer to events that appeared after palivizumab administration or worsened relative to the pre-treatment state, before causality is considered. The treatment-related concept was employed to control for the substantial background influence of symptoms, caused as a result of underlying medical conditions that were unrelated to palivizumab [35]. The AE qualified as a serious adverse event (SAE) if it involved: death, a life-threatening event, hospitalization or prolongation of existing hospital stay, persistent or significant disability or incapacity of the subject, or required medical or surgical intervention to prevent serious outcome [36].

**Fig 1:**
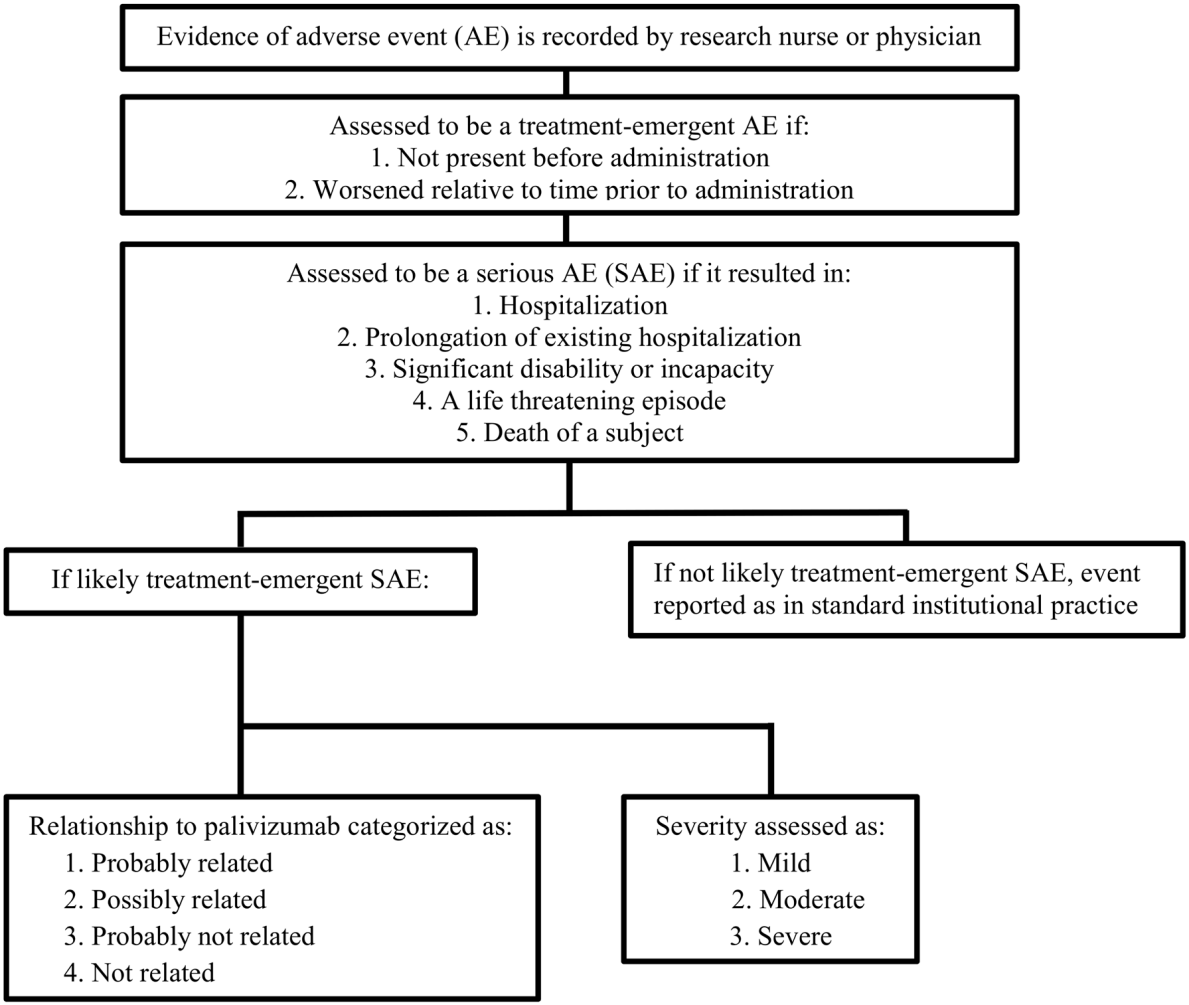
Flow diagram of the assessment of adverse events.

All treatment-emergent AEs that met the criteria of a SAE were submitted to the principal and coordinating investigators within 24 hours of occurrence, using a standardized form. The SAE form was adapted from Health Canada’s AE guidelines to include date of onset, duration, description, severity, outcome, relationship to palivizumab and final diagnosis. The intensity of each event was additionally sub-classified as mild, moderate or severe (Fig 1). A mild event was defined as being transient and easily tolerated; moderate as one causing discomfort with interruption of usual activities and severe as an event with significant interference which may be life-threatening [37].

Upon receipt of a SAE form, the principal investigators of the study, independent of the site investigator, verified the assessment of the relationship of the SAE to palivizumab. Fig 1, summarizes how an adverse event was assessed. The principal investigators assessed the SAE’s relationship to palivizumab using the following definitions: probably related, had a strong temporal relationship to palivizumab administration or recurred on re-challenge and an alternative etiology was far less likely; possibly related, had a strong temporal relationship to palivizumab administration and an alternative etiology was equally or less likely; probably not related, little or no temporal relationship to paliviumab administration or an alternative etiology was more likely; and not related, the event was due to an underlying illness or effect of another drug and was not related to palivizumab (no temporal relationship or more likely alternative etiology exists) [38]. The length of hospitalization was counted as 1 day if any proportion of a day was spent in a defined hospital unit.

In addition, to corroborate physician assessments, the Naranjo Adverse Drug Reaction Probability Scale was used to assess casualty in a standardized way. This scale is based on a list of weighted questions, and an overall score (range: 0–9) evaluates the temporal relationship of the SAE to palivizumab administration. The SAE is then categorized by the score attained as “probably related” (5–8), “possibly related” (1–4), “probably not related or not related” (0) and “unclassifiable” [39].

### Statistical analysis

Data were analyzed by standard descriptive methods using the IBM SPSS statistical software version 20 (IBM Corporation, Armonk, NY). Fisher’s Exact Test was used to examine demographic factors that were potentially different between patients who experienced a palivizumab-related SAE versus subjects in the rest of the database.

## Results

**Fig 2:**
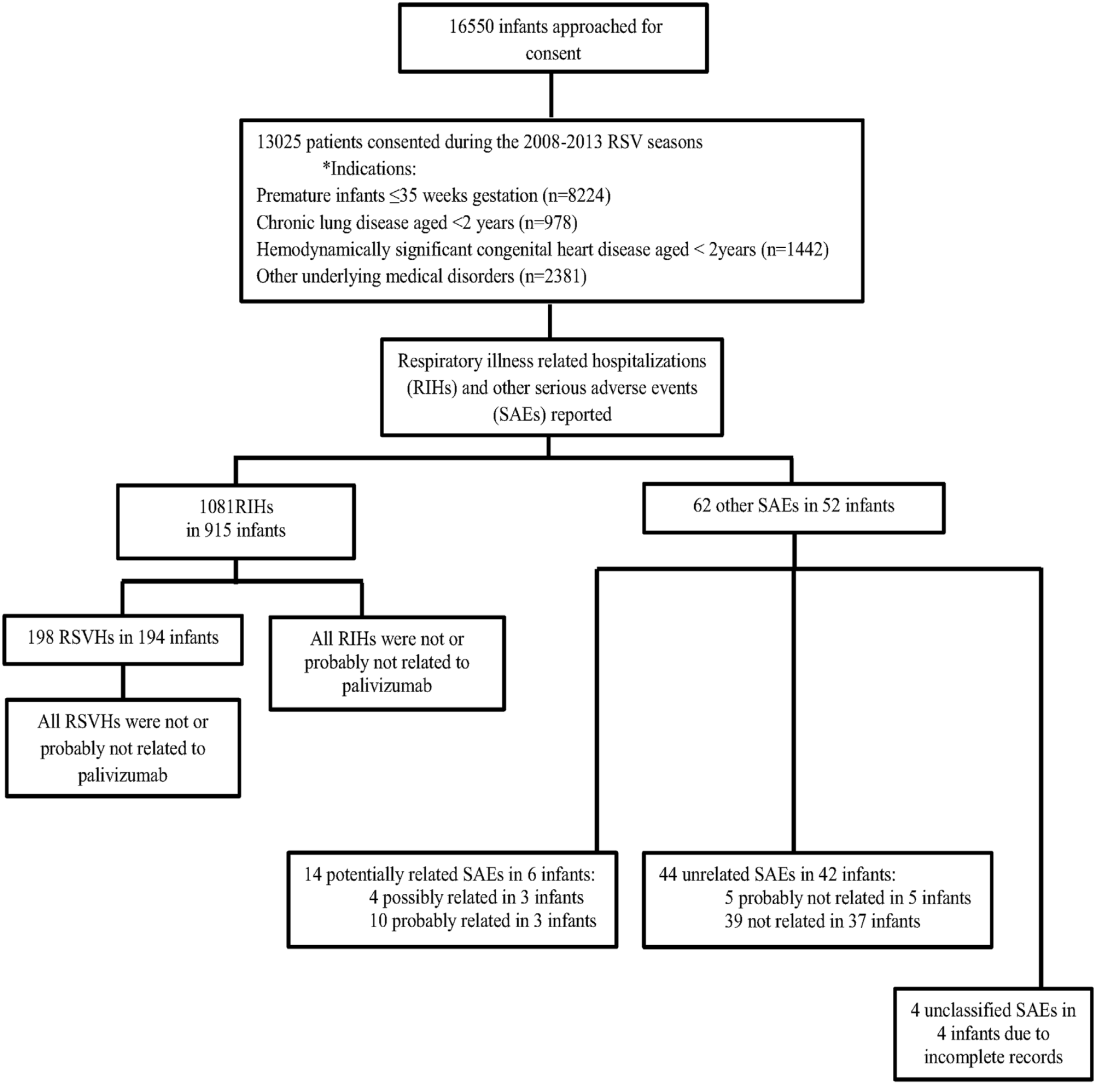
Flow diagram of recruited subjects and related serious adverse events. *Note: The premature group refers to preterm infants only; the “Other” category of infants with serious underlying medical disorders also comprises premature infants.

Over the 2008–2013 seasons, 16550 infants were approached for consent and 13025 (78.7%) infants were enrolled into the CARESS registry (Fig 2). Reasons for consent refusal included, but were not limited to: language barriers, parent/guardian refusal, and inability to be contacted by phone during follow-up. The pooled, consented cohort comprised premature infants ≤35 completed weeks gestational age (n = 8224; 63.1%), children aged <2 years with hemodynamically significant congenital heart disease (HSCHD, n = 1442; 11.1%) and bronchopulmonary dysplasia (BPD, n = 978; 7.5%), and those with pre-existing, complex medical conditions (Other, n = 2381; 18.3%) (Fig 2). The latter group involved patients with Down syndrome (n = 552), congenital airway anomalies (n = 397), pulmonary disorders (n = 358), cystic fibrosis (n = 255), neuromuscular impairments (n = 150), multiple system chromosomal or dysmorphological syndromes (n = 146), immunological compromise (n = 61), serious cardiac conditions beyond 2 years of age (n = 46) and other miscellaneous indications (n = 416). A total of 57392 injections were administered, with a mean (±SD) of 4.3 (±1.4) for prematurity, 4.8 (±1.2) for BPD, 4.5 (±1.4) for HSCHD and 4.6 (±1.3) for those in the “other” category. Over the five RSV seasons, 513 infants (3.9%) received 6 injections and 1.1% (n = 12) received 7 injections, principally because of prolonged RSV seasonality. Subjects were administered an average of 92.7% (±16.1%) of their expected injections, and 75.0% of patients received all of their injections within the recommended time intervals.

### Serious AEs in the CARESS registry

There were 1081 RIHs in 915 patients that were deemed unrelated to palivizumab. Of those, 194 (21.2%) infants tested positive for RSV infection in 198 hospitalizations. Apart from the RIHs, a total of 62 other SAEs reported in 52 infants were submitted for review to the principal investigators of the study. After final assessment, fourteen events in 6 patients (0.05%) were determined to be “possibly related” or “probably related” to palivizumab. Local investigators at each site and subsequently the principal investigators assessed the submitted SAEs and agreed on the same ratings. Forty-four events in 42 patients were categorized as probably not related or not related. Among the 42 patients, 17 had both an unrelated SAE and a RIH. The SAEs that were probably not related and not related (n = 44) included: respiratory distress or failure (n = 13), planned surgeries (n = 9), cardiopulmonary failure due to underlying illness (n = 3), pulmonary infections (n = 2), supraventricular tachycardia (n = 2), sepsis (n = 1), otitis media (n = 1), scarlet fever (n = 1), neurological disorders (n = 1), and other miscellaneous SAE diagnoses (n = 11). The remaining 4 events in 4 patients were deemed unclassifiable as their records were incomplete. The categorization of the 62 reported SAEs is shown in Fig 2. Again, all of the SAEs deemed unrelated (not or probably not related) by each local site investigator were also deemed unrelated by the principal investigators. Five of the 48 SAEs resulted in deaths and were a consequence of pre-existing medical conditions (hemodynamically significant congenital heart disease, complex developmental and/or neurological anomalies, congenital dysmorphological syndromes, and immunocompromise). None of the deaths were associated with RSV infection. The demographics of the population, stratified by AE experience are presented in Table 1.

**Table 1:** Demographics of patients in the CARESS registry by SAE experience (2008–2013).

Characteristics, n (%)	No SAE N = 12075 (%)	RIH (Non-related) N = 915 (%)	RSVH (Non-related) N = 194 (%)	Other Non-Related SAE N = 46 (%)	Related SAE N = 6 (%)
Males	6787 (56.2)	525 (57.4)	107 (54.6)	32 (69.6)	4 (66.7)
Caucasian	8300 (68.7)	649 (70.9)	138 (70.4)	32 (69.6)	3 (50.0)
Daycare attendance	411 (3.4)	59 (6.5)	17 (8.7)	2 (4.3)	1 (16.7)
Atopy in the family	4802 (39.8)	418 (45.7)	98 (50.0)	20 (43.5)	3 (50.0)
Multiple birth	3500 (29.0)	187 (20.4)	59 (30.1)	7 (15.2)	2 (33.3)
Maternal smoking	1612 (13.3)	176 (19.3)	41 (21.0)	7 (15.2)	1 (16.7)
Mother smoked during pregnancy	1570 (13.0)	167 (18.3)	39 (19.9)	5 (10.9)	1 (16.7)
Smoking in the home	3184 (26.4)	297 (32.5)	69 (35.2)	5 (10.9)	1 (16.7)
≥2 smokers in the home	1263 (10.5)	124 (13.6)	27 (13.8)	6 (13.0)	0 (0.0)
Siblings	7651 (63.4)	680 (74.3)	161 (82.1)	30 (65.2)	5 (83.3)
Siblings in daycare	2203 (18.2)	251 (27.4)	62 (31.6)	9 (19.6)	2 (33.3)
≥5 people in the household	3204 (26.5)	284 (31.0)	77 (39.3)	12 (26.1)	3 (50.0)
Mean enrolment age (months ± SD)	5.4 (6.2)	7.9 (8.4)	6.8 (7.9)	6.93 (8.2)	7.9 (8.3)
Mean gestational age (week ± SD)	32.5 (5.9)	33.4 (5.3)	32.8 (5.6)	34.3 (6.8)	32.4 (4.7)
Mean birth weight (g ± SD)	1912 (948)	2088 (1032)	1963 (970)	2199 (907)	1850 (808)
Mean enrolment weight (g ± SD)	5028 (2853)	5914 (3213)	5563 (3451)	5307 (3891)	6015 (3295)

AE, adverse event; SAE, serious adverse event; RIH, respiratory illness-related hospitalization; RSVH, respiratory syncytial virus hospitalization; g, grams; SD, standard deviation.

Please note that the columns are not mutually exclusive (17 patients had a RIH and a non-related SAE, 194 patients had a RSVH that is also a RIH).

### Palivizumab-related SAEs in the CARESS registry

Six patients (0.05%) experienced a total of 14 possibly related or probably related SAEs for an incidence of 2.8 per 10,000 patient-months. Their demographic characteristics did not differ significantly (p >0.05) from the rest of the patients within the CARESS database (Table 1). In four patients, the adverse events had multiple recurrences upon re-challenge with palivizumab, and the remaining two patients had a single SAE. Each of the six patients experienced possible hypersensitivity reactions as verified by the site investigators: 10 events in 3 patients were assessed to be “possibly related” to palivizumab and 4 events in 3 infants were “probably related”. These AEs were verified by the Naranjo scale, with the SAEs of 3 patients categorized as “possibly related” and the SAEs of the other 3 categorized as “probably related” to palivizumab administration. Details of each patient are summarized in Table 2. Symptoms reported in association with the respective SAEs were: erythema or urticaria on the face and body, difficulty swallowing and sucking, vomiting, nasal congestion, bronchospasm and acute respiratory distress (Table 2). All the events resolved after monitoring for 30 days with no immediate life-threatening consequences (Table 2). Two patients were hospitalized: Case 1 was seen in the ER for the first event and was maintained in hospital for 1 day, and Case 4 was admitted for 2 days for monitoring with resolution of the clinical state. The remaining events were categorized as “medically important”. In terms of onset, three of 6 patients had adverse reactions after the first injection (Table 2). All three of these patients had not received palivizumab in a previous RSV season (Table 2; Cases 2, 3, and 5). One patient experienced the SAE after the second injection (Case 6) and another patient experienced it after the third injection (Case 4). One patient was symptom free during the first season while receiving palivizumab, but experienced an allergic reaction following the second injection during the second season (Case 1). Demographic factors (family history of atopy, maternal smoking and smoking during pregnancy, or exposure to smoking in the home) among these 6 patients compared to the rest of the subjects in the database were not significantly different using Fisher’s Exact Test (p>0.05).

**Table 2:** Summary of cases with potentially related serious adverse events (2008–2013).

Case	Age (months)	Indication	Event Description	SAE Criteria	Severity	Relationship with Palivizumab	Naranjo Rating [39]	Total # Events	Re-challenge
1	18	Other; CAA	No AEs: 1^st^ season and 1^st^ dose of 2^nd^ season. Facial + body erythema + bronchospasm after 2^nd^ + 3^rd^ dose of the second season. Possible allergic reaction.	Hospitalization and medically important event	Mild	Probably related	5	2	Yes. Positive re-challenge; erythema on face and body after the third dose.
2	0.7	Premature	Prolonged vomiting and nasal congestion after each dose.	Medically important event	Moderate	Possibly related	6	4	Yes. Positive re-challenge; “increase” in stated symptoms after each re-challenge. Parents declined treatment after the 4^th^ dose.
3	0.7	Premature	Prolonged vomiting and nasal congestion after each dose	Medically important event	Moderate	Possibly related	6	4	Yes. Positive re-challenge; “increase” in stated symptoms after each re-challenge. Parents declined treatment after the 4^th^ dose.
4	2.4	BPD	Generalized urticaria soon after the 3^rd^ dose. Vomiting post-feeds. Hospitalized overnight. Possible allergic reaction.	Hospitalization	Moderate	Probably related	3	1	No
5	7.3	Premature	Facial erythema 5 min. post injection. Patient released after 1 hour.	Medically important event	Mild	Probably related	3	1	No
6	18	HSCHD	Localized rash near injection site after 2nd + 3^rd^ dose.	Medically important event	Mild	Possibly related	4	2	Yes. Positive re-challenge; injection site rash after 3^rd^ dose (re-challenge).

BPD, bronchopulmonary dysplasia; CAA, congenital airway anomalies; HSCHD, hemodynamically significant congenital heart disease.

### Palivizumab discontinuation

Twenty infants discontinued palivizumab due to perceived adverse events after the injections. Nine of the 20 patients experienced an SAE, 6 of which were deemed as possibly (n = 3) or probably (n = 3) related to palivizumab administration (Table 2). Three patients experienced a RIH that was unrelated to palivizumab. The 9 patients are a subset of the overall identified 52 patients who experienced a SAE other than RIHs.

The remaining 11 infants experienced events that did not qualify as SAEs based on defined study criteria. This group comprised: 2 infants who developed rashes on their thighs, 1 infant who experienced a gastrointestinal upset, 3 infants who were “fussy”, 2 were “unwell” post-palivizumab administration, and 3 events were associated with an unspecified AE.

## Discussion

This is the first, prospective, systematic report of SAEs in Canadian infants who received palivizumab for the prevention of RSV infection during the post marketing phase and is the largest study of its kind to date. We were not able to detect any demographic risk factors that were significantly associated with palivizumab-related SAEs. This may either be due to the relatively small number of SAEs related to palivizumab or the idiosyncratic nature of these reactions. The overall number of SAEs found in this cohort was low, but comparable to rates noted in previous studies[20, 25, 27, 29, 31, 32, 40], the majority of which evaluated infants who met regulatory approved indications for prophylaxis universally. Our study is unique, because it also encompasses the largest group of infants (n = 2381, 18.3%) with complex medical disorders who may have a higher risk of SAEs [41, 42] that may result in serious respiratory-related hospitalizations and RSV associated mortality.

The multicenter phase III-IV Expanded Access trial noted that 39 out of 565 patients, who were premature (≤35 weeks gestation) or who were aged <2 years with chronic lung disease, sustained a total of 40 palivizumab-related adverse events. None were severe, 7 (18%) were moderate and 33 (82%) were mild. Few patients (1.9%) withdrew from the study due to adverse events, and 3 out of the 11 withdrawals (27.3%) were due to possibly or probably palivizumab-related adverse events[25]. Similarly, none of the CARESS patients experienced a severe palivizumab-related SAE, although 4 (67%) were categorized as moderate and 2 (33%) were mild. Six of the 20 (30%) withdrawals identified in the CARESS registry were possibly related to palivizumab.

In this study, the majority of SAEs deemed possibly or probably related to palivizumab were hypersensitivity reactions. Monoclonal antibodies such as palivizumab are known to have immunogenic potential and may induce anti-palivizumab antibodies. Initial exposure to the monoclonal antibody sensitizes IgE, which may result in hypersensitivity reactions caused by the IgE-mediated release of inflammatory mediators upon subsequent dosage [43]. In CARESS, three patients experienced hypersensitivity reactions following previous exposure to palivizumab. In particular, one of the three patients reacted following the second injection of their second season of palivizumab therapy, but had no such occurrence in the first season. Lacaze-Masmonteil et al. investigated whether or not there is an increased occurrence of hypersensitivity reactions during the second season of palivizumab and concluded that there were no significant differences compared to the first season, but the sample size was relatively small [20]. This is reflected in our results as the SAEs in the remaining half of the cases (n = 3) occurred following the first injection.

In another study, Lacaze-Masmonteil et al. described 15 adverse events among 516 children that were potentially related to palivizumab administration [28]. While the seriousness of events was not assessed, they were reported as: apnea, fever, injection site pain, hyperventilation, asthenia, vomiting, bronchitis, worsening cough, and urticaria. The symptoms are similar in character to those reported in CARESS, such as erythema, vomiting, nasal congestion, and bronchospasm.

No significant demographic differences (family history of atopy, maternal smoking and smoking during pregnancy, or exposure to smoking in the home) were found between patients who experienced possibly or probably related SAEs and the rest of the subjects in the database. However, this may be due to insufficient analytic power to detect such differences in a small number (n = 6) of patients.

Moore et al. noted that palivizumab accounted for 15% (113/769) of reported deaths to the FDA within a 38 month study period (Nov 1997-Dec 2000), and that palivizumab was suspected in 28% of the cases to be associated with a serious or fatal adverse event [44]. However, a re-analysis of the data by Geskey et al. confirmed that only 2% of the recorded 133 deaths associated with palivizumab occurred in full-term infants without congenital anomalies, and the increased risk of death in the rest of the children was likely due to other causes, rather than palivizumab [45]. Similar relationships with deaths were noted in the CARESS database, and none of the deaths were related to RSV infection. The majority occurred in patients with serious pre-existing medical disorders which likely contributed to their mortality.

There are some limitations of this study. First, for older infants, the CARESS registry does not link patients in their first season of palivizumab therapy to those in subsequent seasons, because patients are allocated new identification numbers every year, which align with privacy recommendations. Information on previous administration would be useful as patients receiving palivizumab could develop an immune response to the monoclonal antibody [46]. However, this limitation applied only to one case possibly-related to palivizumab, where the child was reported to have received palivizumab injections in the previous year without adverse experiences. Second, patients with possible or probable palivizumab-related SAEs were not tested for anti-palivizumab antibodies. Instead, causality assessment relied strictly on patient history, timing of the event, de-challenge and re-challenge clinically. Thus, it is difficult to definitively conclude whether SAEs that resulted in RSV-positive hospitalizations were either due to reduced efficacy of palivizumab (anti-idiotypic response [18, 27]) or defined immunological reactions caused by anti-palivizumab anti-bodies binding to palivizumab. The development of anti-palivizumab antibodies secondary to hypersensitivity reactions can lead to a substantial decrease in the serum concentration of palivizumab. Therefore, some RSV-positive hospitalizations may be attributed to the sub-optimal therapeutic effect of palivizumab, resulting in an underestimated incidence of SAEs deemed related or possibly related to palivizumab. In previous studies[12, 20, 27], significant anti-palivizumab antibody titres consistently >1:80 were not observed and none of the antibody titres reduced the mean, trough palivizumab serum concentrations to below target, inhibitory RSV levels[47]. Similarly, in a recent systematic review, Wegzyn et al. reported an overall low incidence of anti-palivizumab antibodies in patients born prematurely, and those with BPD and HSCHD [46]. Third, there were incomplete data for the 4 patients who were re-challenged with palivizumab after their initial reaction with the injection. While two patients did not appear to have increased severity in their reactions, the other two cases reported increased and prolonged vomiting and nasal congestion after each injection; the most severe reactions were reported after the fourth and last dose with vomiting occurring approximately 48 hours after the injection. These 4 patients all discontinued prophylaxis after the parents and treating physician discussed a possible relationship between the events and palivizumab. However, no diagnostic tests were done to quantify the level of anti-palivizumab antibodies or serum palivizumab concentrations following these reactions.

The CARESS registry does not include a placebo arm, so it is difficult to assess if infants receiving palivizumab had significantly different rates of SAEs compared to a placebo. In the IMpact-RSV randomized controlled trial in infants with congenital heart disease, no significant differences in the number of reported AEs between placebo and palivizumab were detected by the blinded investigators [12, 13]. Immunogenicity studies conducted in the IMpact trial documented that 0.7% of the palivizumab recipients had anti-palivizumab antibodies following the fourth injection, compared to 1.1% found in the placebo group[12]. In addition, although data were collected prospectively by interviewing parents and guardians of the patient, it is often difficult to obtain complete and accurate data. To minimize the impact of this limitation, measures such as cross-referencing findings with patients’ hospital charts was undertaken with patient consent.

## Conclusion

Under active surveillance, a very small proportion of infants who received RSV prophylaxis in the CARESS registry experienced SAEs that were likely linked to palivizumab. These SAEs appeared to be idiosyncratic and were not significantly associated with demographic and environmental risk factors for RSV-related infection. Overall, palivizumab appears to be a safe and well-tolerated prophylaxis for infants at risk of RSV infection.

## Supporting Information

S1 Form Hospitalization form in the event of a hospitalization associated with respiratory illness. (PDF)
