# Supplementary material for: Serious Adverse Events in the Canadian Registry of Children Receiving Palivizumab (CARESS) for Respiratory Syncytial Virus Prevention
Source: PLoS One. 2015 Aug 3;10(8):e0134711. doi: 10.1371/journal.pone.0134711 (PMC4523213; doi:10.1371/journal.pone.0134711)
Supplement: S1 Form — (PDF) [file pone.0134711.s001.pdf]

Hospitalization Form

Form #: ☐ Patient ID: ☐

1. Date of admission (DD-MMM-YYYY): ☐

2. Date of discharge (DD-MMM-YYYY): ☐

3. Criteria for hospitalization (medical reason):

Apnea	O Yes	O No	
Bronchiolitis	O Yes	O No	
Decreased oxygen saturation	O Yes	O No	O Unknown
If Yes, oxygen saturation: ☐ % O Unknown			
Inability to maintain oral intake	O Yes	O No	
Pneumonia	O Yes	O No	
Respiratory arrest	O Yes	O No	
Respiratory distress	O Yes	O No	
Other reason	O Yes	O No	Specify: ☐

4. Discharge diagnosis:

☐

5. Was cystic fibrosis diagnosed? O Yes O No

Before RSV infection? O Yes O No

Presentation: O Newborn screening O Bowel obstruction O Failure to thrive O Respiratory O Combination

Vitamin levels (if measured): ☐

Albumin (if measured): ☐

CF alleles identified: O Yes O No If yes, specify: ☐

6. Number of days in the ICU: ☐

7. Number of days on respiratory support: ☐ ☐ Ongoing

Details of respiratory support: O IPPV O Oscillator O CPAP O Other, specify: ☐

8. Number of days intubated: ☐

9. Was the patient tested for RSV? O Yes O No

If yes, please answer the following:

10. RSV testing result: O Positive O Negative

RSV sampling method used: O Nasal wash O Nasal swab O Nasal aspirate O Auger

Antigen detection method: O Clearview RSV O Culture O DFA O EIA O Rapid screen O ELISA O PCR O Other, specify: ☐

11. Was the patient tested for respiratory viruses (other than RSV)? O Yes O No

If yes, which of the following?

☐ Influenza A	Result:	O Negative	O Positive
☐ Influenza pH1N1	Result:	O Negative	O Positive
☐ Influenza B	Result:	O Negative	O Positive
☐ Parainfluenza	Result:	O Negative	O Positive
☐ Adenovirus	Result:	O Negative	O Positive
☐ Rhinovirus	Result:	O Negative	O Positive
☐ Metapneumovirus	Result:	O Negative	O Positive
☐ Other, specify: ☐	Result:	O Negative	O Positive

If the patient is positive for H1N1, answer the following:

Did the patient receive seasonal influenza vaccine? ○ Yes ○ No

Did the patient receive the H1N1 vaccine? ○ Yes ○ No

Did the patient receive oseltamivir prophylaxis? ○ Yes ○ No

Did the patient receive oseltamivir treatment? ○ Yes ○ No

Did the patient receive pneumococcal vaccine? ○ Yes ○ No

What was the CBC result? ○ Normal ○ Abnormal

What was the lymphocyte result? ○ Normal ○ Abnormal

Was creatinine kinease (CK) done? ○ Yes ○ No

If yes, what was the result? ○ Normal ○ Ab normal

12. Was there a bacterial infection? ○ Yes ○ No

If yes, please specify: ____________

What source: ____________

Was there a bacterial co-infection? ○ Yes ○ No

If yes, please specify: ____________

What source: ____________

13. Is this considered a serious adverse event (SAE)? ○ Yes ○ No

If the patient experienced a serious adverse event* and associated with palivizumab**, please complete an SAE form

**Serious adverse event – death, life-threatening event, hospitalization, prolongation of hospitalization, persistent/significant disability/incapacity, medically important event*

***Strong temporal relationship or recurs on rechallenge, another etiology is unlikely or less likely*
